# Effect of Erdosteine on Cyclosporine Induced Chronic Nephrotoxicity

**DOI:** 10.5812/numonthly.2694

**Published:** 2012-03-01

**Authors:** Carlo Briguori, Gerolama Condorelli

**Affiliations:** 1 Laboratory of Interventional Cardiology and Department of Cardiology, Clinica Mediterranea, Naples, Italy; 2 Department of Cellular and Molecular Biology and Pathology, Federico II University of Naples, Naples, Italy

**Keywords:** Erdosteine, Cyclosporine

Dear Editor,

Drugs and exogenous toxins, including aminoglycosides, amphotericin B, cisplatin, and radiocontrast media, may cause acute kidney injury (1). The manifestations which indicate that damage is occurring are a sudden increase in kidney injury biomarkers (NGAL [neutrophil gelatinase-associated lipocalin], KIM-1 [kidney injury molecule-1], etc.) and kidney failure (creatinine, cystatin C). This implies a requirement for close monitoring of kidney function with eventual dose reduction or even the interruption of drug administration. However, it may eventuate that, even if the source of the damage is stopped, it could still result in permanent kidney failure. One of the major causes of kidney injury driven by different compounds is an increase in the production of reactive oxygen species (ROS), activation of stress kinases and thus apoptosis (2, 3).

In their study “Protective effect of erdosteine on cyclosporine induced chronic nephrotoxicity in rats,” Uz et al. discuss the role of cyclosporine (CsA) in nephrotoxicity. CsA therapy causes dose and time related deterioration of kidney functions by inducing an increase in free radical production (4). The authors describe erdosteine, a mucolytic and anti-inflammatory agent used for the treatment of chronic pulmonary diseases, as having protective effects against CsA-induced nephropathy. This was assessed by evaluating changes in the levels of antioxidant enzymes and nitric oxide (NO) and by the study of kidney morphology through immunohistochemistry. Erdosteine has an antioxidant activity thanks to the presence of two blocked sulfhydryl groups. Erdosteine induced a marked decrease in proximal tubule degenerative changes compared with the CsA group. Moreover, the activity of the anti-oxidant enzyme glutathione peroxidase (GSHPx), increased after CsA + erdosteine treatment. Also increments of malondialdehyde (MDA) and nitric oxide (NO) levels in the rats’ kidneys were prevented by its administration.

Recently the activity of other mucolitics has been proven to result in a reduction of kidney oxidative damage. Our studies have demonstrated that N-acetylcysteine (NAC), a thiol compound classically known as a mucolytic agent, is a potent antioxidant that scavenges a wide variety of oxygen-derived free-radicals and may be capable of preventing acute kidney injury (5, 6). Our in vitro data, using kidney cells, confirmed that NAC may be effective due to its antioxidant properties by preventing contrast-induced renal cell apoptosis. This effect was dose-dependent: indeed, the greater the dose, the larger the cellular benefit. Although still controversial, the protective effects of NAC in preventing contrast-induced acute kidney injury have been observed in several clinical studies and meta-analyses (6, 7).

In conclusion, mucolytic agents seem to be effective in preventing drug–induced nephrotoxicity. Additional studies are however, warranted in order to clarify whether these drugs should be included in our clinical practice.
